# Co-Infection of *Escherichia coli*, *Enterococcus faecalis* and *Chlamydia psittaci* Contributes to Salpingitis of Laying Layers and Breeder Ducks

**DOI:** 10.3390/pathogens10060755

**Published:** 2021-06-15

**Authors:** Huanxin Fang, Hongkun Quan, Yuhang Zhang, Qiang Li, Yihui Wang, Sheng Yuan, Shujian Huang, Cheng He

**Affiliations:** 1 College of Life Science and Engineering, Foshan University, Foshan 528011, China; ixinpro@outlook.com (H.F.); fsyuans@126.com (S.Y.); sjhuang.foshan@163.com (S.H.); 2 Key Lab of Animal Epidemiology and Zoonoses of Ministry of Agriculture and Rural Affairs, College of Veterinary Medicine, China Agriculture University, Beijing 100193, China; Hongkun.Quan@hotmail.com (H.Q.); zhangyuhang@cau.edu.cn (Y.Z.); liqiang5973@163.com (Q.L.); wyhairforce@126.com (Y.W.)

**Keywords:** salpingitis, layers, breeder ducks, *Escherichia coli*, *Enterococcus faecalis*, *Chlamydia psittaci*

## Abstract

Salpingitis is manifested as hemorrhagic follicular inflammation exudations and peritonitis, leading to reduced egg production and high culling of breeder flocks. From 2018 to 2021, increasing salpingitis during egg peak is threatening the poultry industry post-artificial insemination, both in breeder layers and breeder ducks across China. In our study, *Escherichia coli (E. coli), Enterococcus faecalis* *(E. faecalis)* and *Chlamydia psittaci (C. psittaci)* were isolated and identified from the diseased oviducts using biochemical tests and PCR. To identify and isolate pathogenicity, we inoculated the isolates into laying hens via an intravaginal route. Later, laying hens developed typical salpingitis after receiving the combination of the aforementioned three isolates (1 × 10^5^ IFU/mL of *C. psittaci* and 1 × 10^6^ CFU/mL of *E. faecalis* and *E. coli*, respectively), while less oviduct inflammation was observed in the layers inoculated with the above isolate alone. Furthermore, 56 breeder ducks were divided into seven groups, eight ducks per group. The birds received the combination of three isolates, synergic infection of *E. coli* and *E. faecalis*, and *C. psittaci* alone via vaginal tract, while the remaining ducks were inoculated with physiological saline as the control group. Egg production was monitored daily and lesions of oviducts and follicles were determined post-infection on day 6. Interestingly, typical salpingitis, degenerated follicles and yolk peritonitis were obviously found in the synergic infection of three isolates and the birds inoculated with *C. psittaci* alone developed hemorrhagic follicles and white exudates in oviducts, while birds with *E. faecalis* or *E. coli* alone did not develop typical salpingitis. Finally, higher *E. coli* loads were determined in the oviducts as compared to *E. faecalis* and *C. psittaci* infection. Taken together, the combination of *E. coli* and *E. faecalis*, and *C. psittaci* could induce typical salpingitis and yolk peritonitis both in laying hens and breeder ducks. Secondary infection of *E. coli* and *E. faecalis* via artificial insemination is urgently needed for investigation against salpingitis.

## 1. Introduction

Salpingitis has been prevalent both in breeder layers and duck flocks during egg peak since the implementation of an antibiotic reduction program in 2017 in China. Moreover, more frequent cases of salpingitis occur post-artificial insemination and roughly 10–25% breeder birds have been eliminated from flocks due to poor egg production and low fertility. No practical approach is available to control the affected flocks due to lack of knowledge of pathogenesis and virulence factors. In a previous report, duck salpingitis was associated with *Chlamydia psittaci* (*C. psittaci*) infection [1], *Escherichia coli (E. coli)* and *trichomonad* [2], and *Riemerella anatipestifer* (*R. anatipestifer)* [3], while *Mycoplasma anserisalpingitidis (M. anserisalpingitidis)* infection was associated with reproductive diseases in domestic geese [4,5]. Mortality and average condemnation varied from 1.5% in breeding Pekin ducks to 1.98–5% in other breeder ducks, depending on the isolates, mixed infection and virulence of the strains. *R. anatipestifer* and *E. coli* were identified as dominate strains from salpingitis in egg-laying ducks [6]. As for major pathogens, *E. coli* was recovered from a variety of laying hens suffering from a complex of salpingitis, peritonitis and salpingoperitonitis (SPS), and other bacteria, including *staphylococci*, *Mannheimia haemolytica* and *Streptococcus bovis* were isolated from a few carcasses, either alone or together with *E coli* [7,8], *Gallibacteruim anatis(G. anatis)* [9,10], human *uropathogenic E. coli* [11], *G. anatis biovar haemolytica* [12], *Avian influenza virus* H9N2(AIV H9N2) and infectious *bronchitis virus* (IBV). Roughly 3.5% of reproductive tract lesions were observed postmortem, while *E. coli* and *G. anatis* were the major pathogens in laying hens [10]. *E. coli* isolated from salpingitis in broiler parents was found to be transmitted to broilers in which some sequence types contributed to first week mortality of progeny [13]. Recently, AIV H9N2 was replicated in the oviduct of 60% of hens causing different degrees of salpingitis throughout the organ [14]. Salpingitis can either progress from an ascending bacterial infection of the intestinal lumen to the oviduct via the cloaca, or by descending from the respiratory tract (air sac and lungs) [15].

Regarding preventive measures, the putative F17-like fimbrial subunit protein FlfA from *G. anatis* could induce immunization, suggesting the potential of FlfA as a serotype-independent vaccine candidate [16]. Moreover, hens immunized with the recombinant N terminus of *Gallibacterium* toxin A (GtxA-N) generated significantly increased antibody titers against GtxA-N in serum and egg yolk IgY [9]. Autogenous *E. coli* vaccines to prevent *E. coli* peritonitis syndrome in laying hens are often used in the field, and vaccine induced (almost) complete protection against homologous challenge, while protection against heterologous challenge was inconclusive [17]. However, chickens with autogenous *E. coli* vaccine had severe pathological manifestations similar to findings in the unvaccinated group post-challenge with a homologous, as well as a heterologous, *E. coli* strain. Although significantly increasing IgY antibodies were observed in the twice vaccinated group, antibodies did not confer significant protection in broiler breeders [18].

Due to the emergence of widespread multidrug resistance, treating the above bacterial pathogens with traditional antimicrobial drugs is discouraged [19]. Particularly, *G. anatis* isolates were susceptible to apramycin, florfenicol and neomycin and resistant to clindamycin, sulfathiazole and penicillin [20]. Oral administration of the antibiotic doxycycline was found to effectively control the *C. psittaci* infection and recovery of egg production in laying ducks [1]. Oral supplementation of probiotic *Lactobacillus* spp. could prevent duck infection with avian pathogenic *E. coli* (APEC) [21]. In addition to the aforementioned pathogen attacks, stringent biosecurity, regular vaccination, quality feed, clean water, proper ventilation and adoption of a caged-laying housing system, instead of litter-based and free-range systems, are required to minimize infection levels [22].

From 2019 to 2021 in Southern China, the diseased breeder ducks suffered from low egg production and salpingitis was frequently delivered to our lab for diagnosis and control measures. Clinically, egg production was reduced from 85% to 45% in 15 breeder flocks aged 30–35 weeks and some flocks decreased to non-performance in the breeding duck sheds. The affected breeder ducks included several species, such as breeding Pekin ducks, Sheldrake and Muscovy ducks. The present study aimed to isolate and identify the pathogens *E. coli, E. faecalis and C. psittaci* from the diseased ducks. Furthermore, a salpingitis model was developed to elucidate pathogenesis by inoculating isolate alone or synergic infection, both in laying hens and breeder ducks, contributing to strategic prevention of reproductive tract lesions in breeder flocks and to a sustainable poultry industry.

## 2. Results

### 2.1. Seroprevalence of C. psittaci in the Affected Flocks

In the present study, 11 healthy flocks and 15 affected flocks were included in the survey, amounting to 10,500 breeder ducks aged 30–45 weeks. The affected ducks yielded 49.5% and 31.2% eggs during peak period as compared to 82.5% and 85.7% egg performance in the healthy flock. With respect to positive *C. psittaci* antibodies, 64.2% and 85.0% positivity were detected in the Sheldrake and Muscovy flocks in the affected breeder ducks, while 10.0% and 11.6% were determined to be positive in the unaffected flock (Table 1). More interestingly, high chlamydial positivity and low egg performance was detected in the Muscovy flock, indicating a negative correlation between *C. psittaci* infection and egg performance.

### 2.2. Pathogens Isolated from the Breeder Ducks with Salpingitis

**Figure 1:**
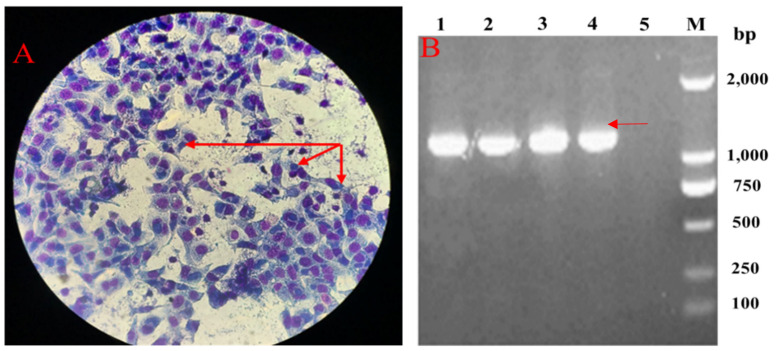
Isolation and identification of duck *C.* *psittaci. (***A**). Chlamydia inclusion bodies in HeLa cells; (**B**). PCR electrophoresis of *ompA* gene (Lines #1–3 *ompA*- pMD19-T vector; Line # 4. Positive control of *ompA* gene; Line #5 Negative control).

**Figure 2:**
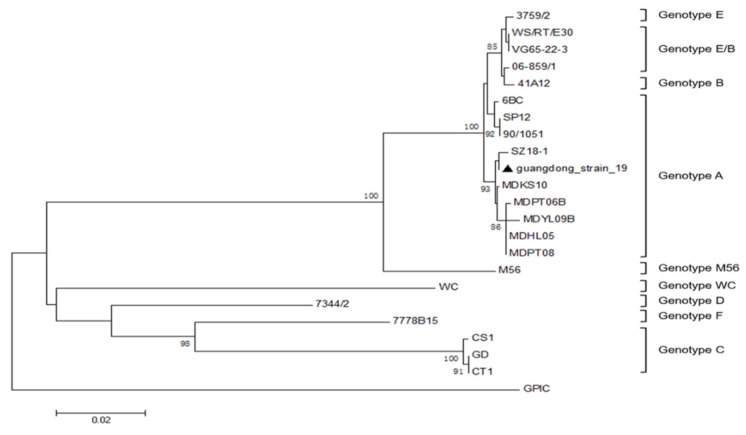
Phylogenetic tree of *ompA* genes of *C. psittaci*, constructed using neighbor-joining method. ▲ The isolate in this study.

Post-inoculation into 7-day-old embryonated chickens, the embryos died between day 3 and day 5 and the mortality occurred regularly after two passages. Postmortem, characteristic vascular congestions of yolk sac were observed in the fetus. Typical inclusion bodies were distributed in Hela cells (Figure 1A). Moreover, DNA extracted from cell cultures produced the expected PCR products using *ompA* gene specific primers, with the size of target gene being approximately 1209 bp, and the recovered PCR products were cloned into plasmid vector and then transformed into competent Dh5a *E. coli* host cells. Positive clones were determined by restricted enzyme digestion and sequenced to confirm the presence of the insert (Figure 1B). The cloned *ompA* gene sequences were compared with the sequences of reference strains and genotype was analyzed by phylogenetic tree. Finally, *ompA* gene of duck isolate was 99.59% and 99.34% identical to that of the *C. psittaci* SZ18-1 strain (GenBank MK751470.1) and *C. psittaci* 6BC strain (GenBank X56980.1), respectively (Figure 2), indicating that *ompA* sequence of the duck isolate was close to the *C. psittaci* avian type A (GenBank accession No. BankIt2452941 Guangdong_strain_19 MZ005959). As for presence of external virus, the chicken embryo chorioallantoic membrane (CAM) was negative for *Newcastle disease virus (NDV)*, *Avian influenza virus (AIV)* and *Infectious bronchitis virus (IBV)* by hemagglutination (HA) and hemagglutination inhibition (HI) assay.

### 2.3. Identification and Propagation of E. coli and E. faecalis

**Figure 3:**
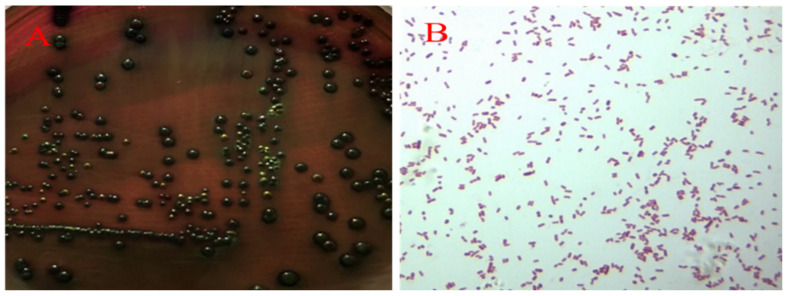
Isolation and identification of *E. coli*. Large black colonies grew on EMB agar (**A**) and Gram-negative short bacillus was observed under microscope (×100) (**B**).

**Figure 4:**
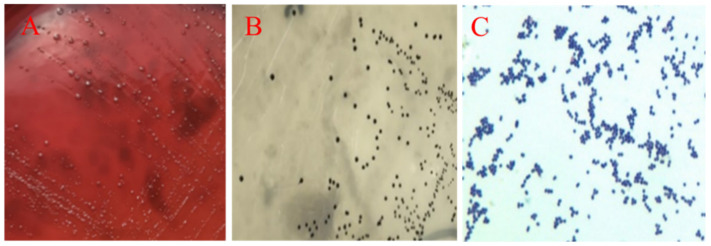
Isolation and identification of *E. faecalis*. White colonies with γ-haemolysis grew on blood agars (**A**) and no transparent rings were observed on Baird-Parker agars (**B**). Gram-positive, chain-forming, and coccus-shaped organisms were visible under microscope (×100) (**C**).

The large colonies grew fast as the black strains on EMB agar (Figure 3A) and the isolate were Gram-negative short bacillus (Figure 3B). Based on the biochemical test and PCR, the isolate was determined to be *E. coli* (GenBank accession No. SUB9521289 QHK2 MW995977). Additionally, grey-transparent to white colonies were observed to develop, circular with an entire margin, convex and surrounded by a pronounced zone of γ-haemolysis on blood agars (Figure 4A), and no transparent rings on the outer side were found on Baird-Parker medium (Figure 4B).Under the microscope, the Streptococcus isolate was found to be a Gram-positive, chain-forming, coccus-shaped organism (Figure 4C) and the isolate was determined as *E. faecalis* by biochemical assays and PCR (GenBank accession No. SUB9498968 QHK1 MW980587).

### 2.4. Salpingitis Induced by E. coli, E. faecalis, C. psittaci or Synergetic Infection in Breeder Layers

**Figure 5:**
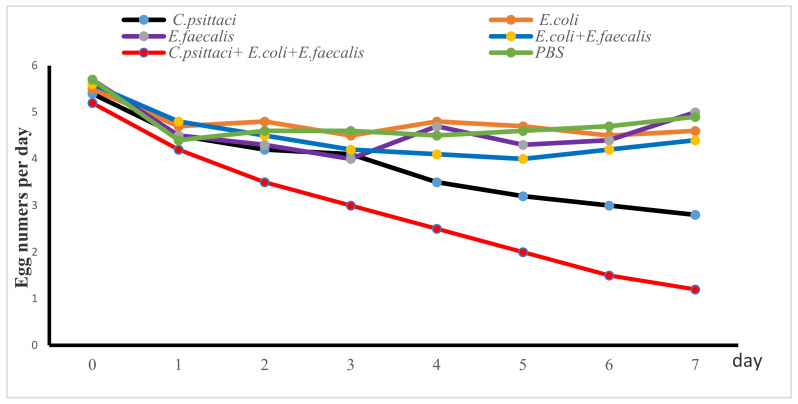
Egg production post inoculation with isolate alone or synergetic infection in breeder layers.

Post simultaneous infection with three pathogens, egg numbers were reduced dramatically while egg drop showed a continuous decline in the breeder hens inoculated with *C. psittaci* alone. More importantly, the combination of three isolates exacerbated egg drop until the last observation. However, breeder hens inoculated with *E. coli* and *E. faecalis* recovered egg production and no significant difference of egg drop was found in comparison with *E. coli* alone or *E. faecalis* alone (Figure 5).

**Figure 6:**
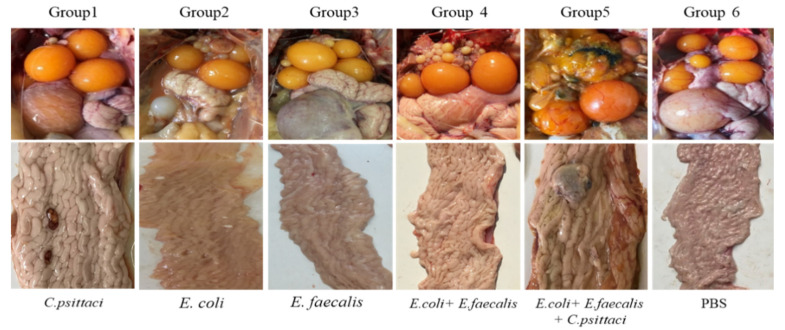
Lesions of follicles and oviducts post inoculation with isolate alone or synergetic infection in layers.

Postmortem, development of secondary ovarian follicle and oviduct inflammations were used to monitor lesion scores. Breeder layers inoculated with *E. coli* and synergetic infection developed follicular deformities and white cheesy-like exudates or blue inflammations in the fallopian tubes. Particularly, 2 peritonitis and slight exudates were observed in the birds inoculated with *E. coli* while five birds developed severe degrative follicles in breeder hens inoculated with the aforementioned three pathogens. As for the birds inoculated with *E. coli* and *E. faecalis*, follicular deformities were evident, accounting for 20% lesions. However, no typical lesions were found in the follicles and oviducts in the birds receiving *E. faecalis* and in the healthy control (Figure 6).

Post cultivation of oviduct tissues in bacterial media or cell cultures, quantities of *C. psittaci*, *E. coli* and *E. faecalis* were determined. Interestingly, higher bacterial colonies were determined in birds with syngertic infection compared to inoculation alone. Similarly, increasing baterial loads were observed both in the *E. coli* and *E. faecalis* group and the combination group with three isolates (Table 2).

### 2.5. Salpingitis Induced by E. coli, E. faecalis, C. psittaci or Synergetic Infection in Breeder Ducks

In the layer model, *C. psittaci* infection alone and the mixed infection with three isolates could both induce egg drop, follicular deformities and inflammation exudates in oviducts. In order to assess whether the isolates were associated with egg reduction, salpingitis and yolk peritonitis in breeder ducks, laying ducks were employed to unveil the pathogenesis. Post-artificial inoculation via intravaginal route, egg numbers were reduced dramatically in the birds inoculated with *C. psittaci,* or *C. psittaci + E. coli* or *C. psittaci + E. coli + C. faecalis*. On the contrary, the birds produced eggs every two days in the ducks inoculated with *E. coli* alone or synergic infection with *E. coli* and *E. faecalis,* and the egg numbers increased to normal situation 5 days later.

**Figure 7:**
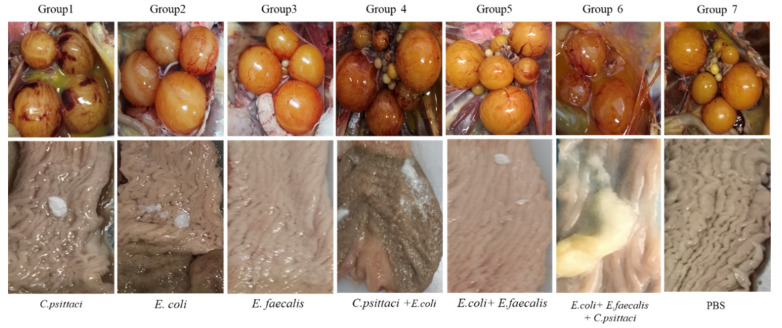
Lesions of follicles and oviducts post inoculation with isolate alone or synergetic infection in breeder ducks.

Postmortem, hemorrhagic follicles and a few white caseous exudates were manifested in the ducks inoculated with *C. psittaci* isolate (Group 1). More interestingly, severe broken follicles and large exudates were observed in the birds infected with the *C. psittaci + E. coli* (Group 5), and the birds infected with *C. psittaci + E. coli + C. faecalis*. Compared to the synergic infection with *C. psittaci + E. coli*, six out of eight ducks developed severe yolk peritonitis and peptone-like exudates with *C. psittaci + E. coli + C. faecalis*. As for ducks infected with *E. coli + E. faecalis*, hemorrhagic inflammations were evident in follicles (Group 4). No typical inflammation was found in the birds infected with *E. coli* alone (Group 2) and *E. faecalis* alone (Group 3) (Figure 7).

Obviously, the isolates were recovered from the fallopian tubes and higher loads were found in the mixed infection compared to the single inoculation. Additionally, severe lesions showed a positive correlation with multi-pathogens while *C. psittaci* inoculation played a dominant role in the pathology of reproductive tracts (Table 3).

## 3. Discussion

### Multi-Agents of Salpingitis during Production Peak of Laying Hens and Breeder Ducks

In the present study, positive Chlamydial antibody showed a negative correlation with the reduced egg production in breeder ducks, accounting for six–eight-fold seropositivity compared to unaffected duck flocks. Subsequently, *E. coli*, *E. faecalis* and *C. psittaci* were isolated and identified from fallopian tubes of the diseased breeder ducks by cultivation, biochemical test and PCR assay. Post-artificial infection with above isolate alone or combination, *C. psittaci* infection alone and combination with other two agents might contribute to degenerative follicles and salpingitis, both in layers and breeder ducks, suggesting that *C. psittaci* infection might trigger the first attack wave and secondary infection by *E. coli*, and *E. faecalis*. Compared to *C. psittaci* infection alone, breeder ducks infected with three isolates developed severe raptured follicles, extensive exudates in fallopian tubes and yolk peritonitis, leading to reduced egg production and high culling during the egg peak period, which correlates with clinical inflammation in the affected duck flocks.

In the field study, salpingitis is prevalent both in layer flocks and breeder ducks, which hinders sustainable poultry development. The main causes of salpingitis in duck flocks might be associated with the following factors. (1) Artificial insemination procedures facilitate chlamydial dissemination from male birds to female birds and cross-infection among the female flock. *C. psittaci* is reported to infect other birds via vertical route [23,24,25,26] and horizonal transmission [27,28]. Moreover, Chlamydia infection might be associated with estrogen secretions during peak production. The age-related plasma hormone measurements in a previous study revealed a relationship between changes in hormone levels and age at puberty. Estrogen concentration at peak showed a correlation with egg production [29]. In order to develop an animal model of human fallopian tubes, mice were injected subcutaneously with 0.5 mg of estradiol valerate 72 h prior to *Chlamydia trachomatis* via intravaginal inoculation [30,31,32,33]. During peak production, highly concentrations of estrogen will facilitate *Chlamydia* infection, indicating that high seroprevalence is associated with Chlamydia infection in breeder flocks. In the previous report, *Chlamydia* can be transmitted horizontally through the fecal–oral route, and the incidence of ovarian inflammation might be associated with *Chlamydia* infection. In addition, *C. psittaci* was transmitted vertically through eggshell penetration [28]. On other hand, semen from male ducks (drakes) might be a novel source of Chlamydia infection. In our routine test, an average 40% positive Chlamydial inclusions were identified from the semen of the affected duck flocks. Particularly, artificial insemination procedures are implemented in large scale poultry farms in Southern China. Healthy female flocks will be infected by the contaminated semen due to the absence of an external pathogen test. (2) *E. faecalis* isolates might be associated with large abuse of fermented feedstuff, feeds and probiotic abuse in the poultry industry. Since 2020, antibiotics have been banned in animal feeds across China according to new regulations. On other hand, fermented feedstuff and probiotics are recommended for use in animal diets and probiotics are claimed to replace antimicrobial agents to control infectious diseases. Due to no maximum limitation of probiotics, co-infection of *E. faecalis* and *Ornithobacterium rhinotracheale* (ORT) was reported in lungs, livers and spleens, leading to dyspnea, hemorrhagic discharge in trachea and bronchial obstruction in the broiler industry [34]. (3) It is unclear why three pathogens interact to induce salpingitis both in laying hens and in breeder ducks. As for the opportunistic pathogens, both *E. faecalis* and *E*. *coli* are widely present in the environment and they are diffused by air, water and other means. Traditionally, both pathogens are colonized in gastrointestinal tracts and then transmitted to blood circulation and reproductive tracts once birds suffer immune suppression or external stress. In our previous reports, virulent *C. psittaci* infection induces immunity suppression and triggers secondary infection of avian influenza virus H9N2 or ORT [35]. In this study, synergetic infection might facilitate pathogen survival by increasing bacterial loads in fallopian tubes compared to single infection. Upon *C. psittaci* infection, birds suffer immune suppression due to first attack by Chlamydia infection, and secondary infection by *E. coli* and *E. faecalis* might trigger inflammations in follicles and oviducts, leading to severe exudates and salpingitis in breeder ducks.

In a previous report, duck salpingitis was associated with *C. psittaci* infection [1], *E. coli* and *Tetratrichomonas sp*. [2], *M. anserisalpingitidis* [5,36], and *R. anatipestife* [4]. Moreover, reproductive disorders in layers were associated with infections of *E. coli* [37], *Staphylococci*, *Mannheimia haemolytica*, *Streptococcus bovis* [7], *G.anatis* [9,10], *G. anatis biovar haemolytica* [38]. A recent report stated that A-G1-lineage H9N2 virus with oviduct tropism was responsible for chronic pathological changes in the infundibulum and a long-lasting drop in egg production. Moreover, *E. coli* and *G.anatis* were the major pathogens causing reproductive tract lesions in laying hens [10]. In our study, both layers and breeder ducks inoculated with *E. coli* alone did not develop typical salpingitis as in previous reports [13]. The main reason that *E. coli* isolate from the diseased breeder ducks might be an opportunistic pathogen is its conversion into avian pathogenic agent by co-infection with *C. psittaci* and *E. faecalis*. In the present study, higher bacterial colonies were determined in the synergic infection, indicating that *E. faecalis* promoted *E. coli* infection. Although several types of *E. coli* were reported to be associated with extra-intestinal infections in poultry, the clinical outcome of infection with *E. coli* in poultry is largely influenced by the specific strain as well as individual host factors [8]. In previous records, *E. facelis* infection was associated with septicemia and endocarditis. Clinically, *E. facelis* causes severe acute respiratory distress, sometimes in parallel with high morbidity and high mortality. Chickens infected with *E. facelis* contributed widely to fibrinous exudate and hemorrhagic pneumonia, resulting in occlusion of the alveolar spaces in the presence of ORT primary infection [34]. The presence of *E. facelis* in duck’s oviducts might be associated with its contamination in probiotics since the implementation of an antibiotic reduction campaign in China since 2017.

As for *C. psittaci* isolates, serovar A was endemic among psittacine birds, and serovar B was endemic among pigeons and turkey. Serovar C was isolated from ducks, white swans and partridges. Serovar D was isolated from turkeys, seagulls and budgerigars and serovar E isolate was identified from human pneumonitis. MN isolates have been obtained from ducks, pigeons, ostriches and rheas while serovar F isolate was obtained from a parakeet [39]. In our study, the duck isolate was identified to be *C. psittaci* genotype A and our report was consistent with the previous duck isolate, manifested as low egg production, severe hemorrhagic inflammation in livers and hearts and necrosis of the ovarian follicular tissues [1], indicating that *C. psittaci* genotype A was a circulating strain in the diseased breeder flocks. Regarding pathogenicity of *C. psittaci*, genotype C isolated from hens targeted fallopian tubes to induce cystic oviducts and low egg production in laying hens. However, no degenerative or ruptured follicles were observed during necropsy. The different type of lesion might be associated with different pathogenicity of the genotype C isolate [40]. Based on the above evidence, this is the first time that avian salpingitis model has been developed post inoculation with *C. psittaci* via the intravaginal route. More importantly, egg drop occurred post-inoculation with the aforementioned three isolates. Postmortem, the lesions were characterized as severe salpingitis and degenerative follicles, both in layers and breeder ducks. In an artificial experiment, *C. psittaci* infection played a dominant role in the mixed infection by triggering immune suppression and secondary infection of *E. facelis,* and *E. coli* exacerbated follicle lesions and extensive exudates, leading to yolk peritonitis and large condemnation from breeder flocks. Our study also confirmed the previous salpingitis by mixed infection with *R. anatipestifer, Pasteurella multocida* ssp., *E. coli* and taxon3 in ducks and geese [6]. Additionally, our study imitated artificial insemination during breeder flocks via intra-vaginal inoculation of the isolated pathogens. Compared to the intramuscular route or oral administration in duck models, peritonitis and tubal blockage were similar to clinical lesions with follicular ruptures and necrosis, indicating that hygiene of artificial insemination might contribute to salpingitis by multi-infection with ascending bacteria. Hygienic measures are required to control external pathogens in semen, such as semen contamination, via tube sterilization and semen collection skills.

In summary, our study showed that synergetic infection with three isolates from diseased ducks was able to induce egg production, ruptured follicles, salpingitis and saplings-peritonitis both in layers and breeder ducks. During artificial insemination, *C. psittaci* genotype A triggered hemorrhagic follicles and oviduct exudates while *E. coli* and *E. faecalis* attacked follicles to induce initial ruptured follicles and secondary salpingo-peritonitis, leading to high culling from breeder flocks and huge economic loss. Regarding comprehensive controlling of salpingitis, a vaccination program against *C. psittaci* infection is highly recommended for breeder layers and breeder ducks prior to peak period, while feed-borne *E. facelis* and hygienic measures in artificial insemination are urgently needed. Our data provided an insight into a salpingitis model in breeder flocks and shed light on comprehensive control measures in vaccine development and therapeutic drugs. However, the crosstalk among the three pathogens remains elusive and further investigation is required to unveil the pathogenesis.

## 4. Materials and Methods

### 4.1. Seroprevalence of C. psittaci Infection in the Affected Breeder Ducks

In the present study, a total of 10,500 breeder ducks aged 30–40 weeks were examined in the survey, including 11 healthy flocks and 15 diseased flocks during peak period. Clinically, both Sheldrake and Muscovy ducks suffered from 10–15% egg drop during 30 to 40 weeks, characterized as average 55% egg production, 84% fertility and 30% culling rate from December 2018 to February 2020. In order to determine positive *C. psittaci* infection,10 blood samples were collected from each flock and *C. psittaci*-specific antibodies were determined using MOMP-ELISA kit (Chlamydia ELISA rMOMP (a testing kit for chickens, developed in-house and supplied by Dr. Daisy Vanrompay at Department of Molecular Biotechnology, Faculty of Bioscience Engineering, Ghent University, Belgium) as previous described. The current study was approved both by the Institutional Animal Care and Use Committee (IACUC) at Foshan University and China Agricultural University (Code: IACUC 20190305) and the artificial infections were carried out in an approved animal biosafety level 2 facility at Foshan University.

### 4.2. Chlamydial Isolation and Identification

Chlamydia isolation: the uterine mucosa and oviducts were aseptically obtained from diseased breeder ducks. The tissues were divided into two samples. One sample was minced and treated with Eagle’s minimal essential medium (MEM) containing Gentamycin (200 g/mL) and Vancomycin (1 mg/mL). The samples were vortexed with 3 mm-diameter glass beads, centrifuged at 500× *g* for 5 min at 4 °C and the supernatants (0.4 mL) were collected and injected into yolk sacs of SPF embryonated chickens aged 7 days, 0.2 mL per egg. Afterwards, the embryonated chickens were incubated at 37 °C for one week and the embryonated chickens were monitored twice per day (PDR-1000AN Monitor, Thermo Fisher Scientific, Beijing, China). The second passage was carried out to observe pathogenicity by inoculating with the collected yolk membranes. Meanwhile, embryonated chorioallantoic membranes (CAM) were collected to identify the presence of *Avian influenza virus (AIV)*, *Newcastle disease virus (NDV)* and infectious *bronchitis virus (IBV)* using hemagglutination (HA) and hemagglutination inhibiting (HI) tests (China Institute of Veterinary Drug Control, Beijing, China). Another sample was inoculated into Hela cells at 37 °C in the presence of 5% CO_2_ for 72 h. Afterwards, cell cultures were harvested and purified by density-gradient centrifugation. Purified EBs were quantified following direct Wright-Giemsa Staining (Solarbio Sciences & Technology Co., Ltd., Beijing, China), then stored in sucrose phosphate glutamate (SPG) for further tests.

Chlamydia genotype: DNA was extracted by commercial kit (Sigma-Aldrich, Beijing, China). Firstly, Chlamydia 23S rRNA and *incA* gene were determined using real-time quantitative polymerase chain reaction (q-PCR) (Applied Biosystems™ 7500, Thermofisher, Beijing, China). The q-PCR reagents included 10 μL of 2× AceQ Universal U, Probe Master Mix V2 (Vazyme, Nanjing, China), 2 μL of DNA template, 0.4 μM of specific primers, 0.2 μM TaqMan Probe, and ddH_2_O. Thermal cycling parameters were performed as follows: 37 °C for 2 min; 95 °C for 5 min; 40 cycles of 95 °C for 10 s, 60 °C for 30 s. Secondly, the *ompA* gene of *C. psittaci,* approximately 1209 bp, was amplified and its products were separated on agarose gel and purified using a QIAquick Gel Extraction Kit (Qiagen, Beijing, China).The PCR reagents contained 10 μL of 2× Premix Taq (Ex Taq Version 2.0) (Takara, Beijing, China), 2 μL of DNA template, 1 μM of specific primers, ddH_2_O up. The purified PCR products were cloned with pMD™19-T vector (Takara, Beijing, China) and then sequenced by a commercial company (BGI Genomics Co. Ltd., Shenzhen, China). The other 24 sequences of *ompA* genes *C. psittaci* were downloaded from GenBank, aligned by MEGA (version 7.0) ClustalW algorithm and the Test Neighbor-Joining Tree for designing the *ompA* phylogenetic tree. To evaluate evolution of *ompA* sequences, 500 bootstraps were calculated and the rooted tree was based on *ompA* sequence of *Chlamydia caviae* (GPIC). Moreover, the amplification was performed using universal primers (16S rRNA-27F: 5′-AGAGTTTGATCCTGGCTCAG-3′ and 16S rRNA-1492R: 5′-TACGGCTACCTTGTTACGACTT-3′ (Sangon Biotech Co., Ltd., Shanghai, China) targeting 1500 bp for bacteria. The PCR was carried out at a final volume of 50 µL containing 5 µL 10× PCR buffer, 4 µL dNTPs, 2 µL of each primer, 1 µL rTaq polymerase, 2 µL DNA template and ddH_2_O was added 34 µL. PCR condition were as follows: one pre-denaturation cycle at 94 °C for 5 min, 30 cycles of denaturation at 94 °C for 45 s, annealing at 55 °C for 45 s, elongation at 72 °C for 2 min and one post-elongation cycle at 72 °C for 10 min. PCR products were visualized in 1.20% agarose gel stained with ethidium bromide under UV transillumination, purified by agarose gel recovery kit (Sigma-Aldrich, Beijing, China), and then analyzed by 16S rRNA sequencing (Sangon Biotech, Co., Ltd., Shanghai, China). Multiple sequence alignment and homology analysis were carried out by NCBI/BLAST/blast software and Clustal X software. Chlamydia-specific primers are listed in Table 4 and *ompA* reference sequences described in Table 5.

### 4.3. Identification and Propagation of E. coli and E. faecalis

Postmortem examination, oviduct tissues grew onto standard I nutrient agar (Merck, Berlin, Germany) with 5% sheep blood and were incubated at 37 °C under aerophilic conditions for 24–48 h. The positive colonies were transferred into eosin-methylene blue (EMB) media and Baird-Parker medium (Aoboxing Bio-Tech Co., Ltd., Beijing, China). Afterwards, the typical colonies were identified by Gram staining and biochemical assays (Sigma-Aldrich, Beijing, China). The biochemical tests were determined by BD Phoenix™ automated identification and susceptibility testing system (BD, Beijing, China). Bacterial CFUs were quantified on nutrient agar containing 5% sheep blood.

### 4.4. Experimental Infection in Breeder Layer

Forty Lohman breeder layers aged 180 days were randomly assigned to 5 groups, 8 birds per group and maintained in negative isolators in biosafety level 2 facilities. Group 1 layers were infected intra-vaginally with 0.1 mL of 10^5^ IFU of *C. psittaci* isolate. Group 2 layers were inoculated intra-vaginally with 1.0 × 10^5^ CFUs of *E. coli* isolate. Group 3 layers received intra-vaginally 1.0 × 10^5^ CFUs of *E. faecalis.* Group 4 layers received 1.0 × 10^5^ CFUs of *E. coli* isolate and the same dose of *E. faecalis* via intra-virginal route at the same time while Group 5 hens were inoculated with 0.1 mL of 10^5^ IFU of *C. psittaci*, 1.0 × 10^3^ CFUs of *E. coli* and *E. faecalis* via intra-vaginal route. Group 6 hens received 0.1 mL of sterile PBS via the same route as the healthy control. Clinical signs/symptoms and egg production were recorded twice per day until the end of the experiment. After 7 days, the hens were anesthetized by lethal intraperitoneal injection with 90 mg/kg of sodium pentobarbital and sacrificed to observe lesions of follicles and oviducts. Afterwards, oviduct tissues were collected aseptically to grow onto bacterial media or cell cultures, 5 samples per group.

### 4.5. Experimental Infection in Breeder Ducks

Fifty-six healthy breeder sheldrakes aged 200 days were purchased from a commercial company (Wens Food Co. Ltd., Guangdong, China) and the birds were randomly assigned to 7 groups, 8 birds per group. Group 1 ducks were infected intra-vaginally with 0.1 mL of 10^5^ IFU of *C. psittaci* isolate. Group 2 ducks were inoculated intra-vaginally with 1.0 × 10^5^ CFU of *E. coli* isolate. Group 3 ducks were inoculated intra-vaginally with 1.0 × 10^5^ CFU of *E. faecalis.* Group 4 birds received 1.0 × 10^3^ CFU of *E. coli* isolate and same dose of *E. faecalis* via intra-vaginal route at the same time. Group 5 birds received 0.1 mL of 10^5^ IFU of *C. psittaci*, 1.0 × 10^3^ CFU of *E. coli* via intra-vaginal route. Group 6 ducks received 0.1 mL of 10^5^ IFU of *C. psittaci*, 1.0 × 10^3^ CFU of *E. coli* and *E. faecalis* via the same route. Group 7 birds received 0.1 mL of 0.1 mL of sterile PBS as the control group as above mentioned and all the ducks were kept in individual isolators. Clinical signs/symptoms and egg production were monitored twice per day until the end of the experiment. After 7 days, the ducks were anesthetized by lethal intraperitoneal injection with 90 mg/kg of sodium pentobarbital. Postmortem, follicular deformities and inflammation exudates were observed while bacterial loads were determined in the oviduct samples, 5 birds per group as above described.

## Figures and Tables

**Table 1:** Chlamydia-specific IgG using MOMP-ELISA in breeder ducks.

Breeder Ducks	Clinical Health Flock		Suspected Flock	
	Sheldrake	Muscovy	Sheldrake	Muscovy
Flocks	5	6	7	8
No. breeder ducks	2100	2400	2800	3200
Egg production (%)	82.5	85.7	49.5	31.2
No. blood samples	50	60	70	80
No. positive samples	5	7	45	68
No. negative samples	45	53	25	12
Positive (%)	10.0	11.6	64.2	85.0

**Table 2:** Bacterial loads of oviducts in breeder layers post inoculation.

Group	Inoculates	*Chlamydia* (IFU/g)	*E. coli* (CFU/g)	*E. faecalis* (CFU/g)
1	*C. psittaci*	1.0 × 10^5^	--	--
2	*E. coli*	--	1.0 × 10^6^	--
3	*E. faecalis*	--	--	1.0 × 10^4^
4	*E. coli + E. faecalis*	--	1.1 × 10^8^	1.0 × 10^8^
5	*C. psittaci + E. coli + E. faecalis*	0.7 × 10^5^	2.0 × 10^8^	2.1 × 10^8^
6	PBS	--	1.0 × 10^2^	--

--, No bacteria isolated from oviducts. PBS: phosphate buffered saline.

**Table 3:** Bacterial loads post-inoculation with the isolate alone or mixed pathogens.

Group	Inoculates	*Chlamydia* (IFU/g)	*E. coli* (CFU/g)	*E. faecalis* (CFU/g)
1	*C. psittaci*	2.5 × 10^5^	--	--
2	*E. coli*	--	2.3 × 10^6^	--
3	*E.faecalis*	--	--	1.5 × 10^6^
4	*E. coli + E. faecalis*	--	1.2 × 10^8^	2.6 × 10^7^
5	*E. coli* + *C. psittaci*	3.1 × 10^6^	1.2 × 10^8^	--
6	*C. psittaci + E. coli + E. faecalis*	1.3 × 10^6^	2.0 × 10^8^	1.1 × 10^8^
7	PBS	--	2.2 × 10^2^	--

--, No bacteria isolated from oviducts.

**Table 4:** Chlamydia-specific primers.

Primers	Sequence (5′→3′)	Products	References
23S rRNA	CTGAAACCAGTAGCTTATAAGCGGT ACCTCGCCGTTTAACTTAACTCC FAM-CTCATCATGCAAAAGGCACGCCG-TAMRA	111 bp	Ralf Ehricht et al., 2006 [41]
*C. psittaci*-*incA*	GCCATCATGCTTGTTTCGTTT CGGCGTGCCACTTGAGA FAM-TCATTGTCATTATGGTGATTCAGGA-TAMRA	74 bp	Vogler BR, Trinkler M, et al., 2019 [42]
*ompA*	ATGAAAAAACTCTTGAAATCG TTAGAATCTGAATTGAGCYTTCATYT	1209 bp	Smith KA, Bradley KK et al., 2005 [43]

**Table 5:** *OmpA* reference sequences used in this study.

Strain	Genotype	District	Host	GenBank No.
MDHL05	A	Taiwan, China	Muscovy duck	MK032046.1
MDYL09B	A	Taiwan, China	Muscovy duck	MK032048.1
MDPT06B	A	Taiwan, China	Muscovy duck	MK032049.1
MDPT08	A	Taiwan, China	Muscovy duck	MK032050.1
MDSK10	A	Taiwan, China	Muscovy duck	MK032051.1
SZ-18-1	A	China	Duck	MK751470.1
CS1	C	China	Duck	EU009493.1
CAU1	A	China	Duck	EU101714.1
CAU2	A	China	Chicken	EF202608.1
SP12	A	China	Bird	EU856032.1
06-859/1	E/B	France	Duck	EU159263.2
WS/RT/E30	E/B	Germany	Duck	AY762613.1
GD	C	Germany	Duck	AF269261.1
VG65-22-3	E/B	Germany	Duck	EU019091.1
90/1051	A	Belgium	Parrot	AY762608.1
6BC	A	America	Parrot	X56980.1
41A12	B	Belgium	Turkey	AY762609.1
3759/2	E	Italy	Pigeon	AY762611.1
M56	M56	America	Hares and muskrats	AF269268.1
WC	WC	America	Cow	AF269269.1
7344/2	D	Italy	Pigeon	AY762610.1
CT1	C	America	Turkey	AF269260.1
7778B15	F	Belgium	Turkey	AY762612.1
GPIC	*Chlamydia caviae*	America	Guinea pig	AF269282.1

## Data Availability

The data presented in this study are available on request from the corresponding author and isolate sequnces can be found in GenBank.
